# Modification of MCF-10A Cells with Pioglitazone and Serum-Rich Growth Medium Increases Soluble Factors in the Conditioned Medium, Likely Reducing BT-474 Cell Growth

**DOI:** 10.3390/ijms13055607

**Published:** 2012-05-10

**Authors:** Boon Yin Khoo, Noorizan Miswan, Prabha Balaram, Kalpanah Nadarajan, Elena Elstner

**Affiliations:** 1 Institute for Research in Molecular Medicine (INFORMM), Universiti Sains Malaysia, 11800 USM, Penang, Malaysia; E-Mails: noorizan_miswan@usm.my (N.M.); prabhabalaram@yahoo.co.in (P.B.); kalpanahnadarajan@hotmail.com (K.N.); 2 Division of Oncology and Haematology, Charité Campus Mitte, Humboldt University of Berlin, 10117 Berlin, Germany; E-Mail: elena.elstner@charite.de

**Keywords:** adhesive and non-adhesive cell interaction, cell growth, human breast cancer, pioglitazone, serum-rich growth medium, tumourigenic and non-tumourigenic cells

## Abstract

In the present study, we aimed to preincubate MCF-10A cells with pioglitazone and/or serum-rich growth media and to determine adhesive and non-adhesive interactions of the preincubated MCF-10A cells with BT-474 cells. For this purpose, the MCF-10A cells were preincubated with pioglitazone and/or serum-rich growth media, at appropriate concentrations, for 1 week. The MCF-10A cells preincubated with pioglitazone and/or serum-rich growth media were then co-cultured adhesively and non-adhesively with BT-474 cells for another week. Co-culture of BT-474 cells with the preincubated MCF-10A cells, both adhesively and non-adhesively, reduced the growth of the cancer cells. The inhibitory effect of the preincubated MCF-10A cells against the growth of BT-474 cells was likely produced by increasing levels of soluble factors secreted by the preincubated MCF-10A cells into the conditioned medium, as immunoassayed by ELISA. However, only an elevated level of a soluble factor distinguished the conditioned medium collected from the MCF-10A cells preincubated with pioglitazone and serum-rich growth medium than that with pioglitazone alone. This finding was further confirmed by the induction of the soluble factor transcript expression in the preincubated MCF-10A cells, as determined using real-time PCR, for the above phenomenon. Furthermore, modification of the MCF-10A cells through preincubation did not change the morphology of the cells, indicating that the preincubated cells may potentially be injected into mammary fat pads to reduce cancer growth in patients or to be used for others cell-mediated therapy.

## 1. Introduction

The peroxisome proliferator-activated receptor-gamma (PPARγ) ligands, such as pioglitazone, have been demonstrated to have therapeutic value for human breast cancer treatment [1–3]. Although pioglitazone has been demonstrated not to induce apoptosis in both estrogen receptor (ER)-positive (MCF-7) and ER-negative (MDA-MB-231) tumourigenic human breast cells, as determined in ours (data not shown) and other studies [4], the use of pioglitazone in human breast cancer research remains valuable to be investigated. According to previous studies, pioglitazone demonstrates anti-metastatic effects on the highly invasive tumourigenic human breast cells, MDA-MB-231, by targeting the cells invasive behaviour [1]. Moreover, pioglitazone, as well as rosiglitazone, also have better toxicity profiles than other thioglitazones (TZDs), as determined in our present study (data not shown). However, the effects of pioglitazone on non-tumourigenic human breast cells, such as MCF-10A cells, have not yet been demonstrated. MCF-10A cells may also respond differently to the pioglitazone in serum-rich growth medium. Indeed, the interaction of the MCF-10A cells preincubated (modified) with pioglitazone and/or serum-rich growth media and then co-cultured with tumourigenic human breast cells, such as MDA-MB-231 and BT-474, has never been demonstrated previously.

**Figure 1:**
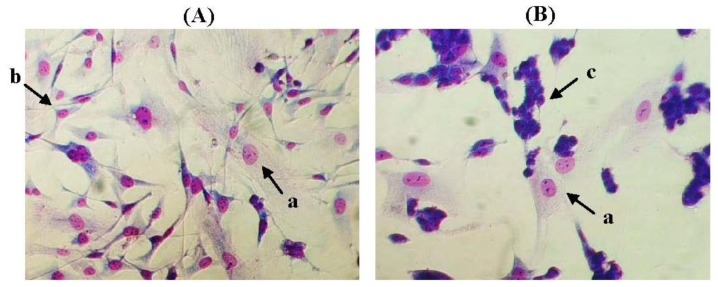
Co-culture of (**A**) MDA-MB-231 cells with MCF-10A cells, and (**B**) BT-474 cells with MCF-10A cells. The BT-474 cells can easily be distinguished from MCF-10A cells, whereas the epithelial-like morphology of MDA-MB-231 cells is not easily distinguishable. The pictures were taken at 40% cell confluence with 200× magnification, where 0.01 mm was equal to 10 μm. **a**: MCF-10A cells, **b**: MDA-MB-231 cells and **c**: BT-474 cells.

MCF-10A cells, which are derived from an adherent human breast cell population of a Caucasian patient carrying fibrocystic disease, are a non-tumourigenic human breast epithelial cell line. The cells are widely used for breast cancer research as a control in comparison to tumourigenic human breast cells mainly due to the cells’ poorly-differentiated and hormone-independent characteristics [5]. In the present study, the adhesive and non-adhesive interactions of pioglitazone and/or serum-rich growth media preincubated MCF-10A cells with BT-474 cells were determined. The study aimed to (1) preincubate MCF-10A cells with pioglitazone and/or serum-rich growth media, and (2) determine the adhesive and non-adhesive interactions of the above preincubated MCF-10A cells with BT-474 cells after pioglitazone and/or serum were removed from the growth media. The soluble factors secreted by the MCF-10A cells that were preincubated with pioglitazone and/or serum-rich growth media were also examined in this study. These soluble factors are believed to be proliferation-related growth factors. Expression of the soluble factors was then confirmed at the transcript level in the preincubated MCF-10A cells. It has been hypothesised that the preincubated MCF-10A cells might behave differently in co-culture with BT-474 cells compared with the non-preincubated MCF-10A cells. BT-474 cells were used instead of MDA-MB-231 in this study because BT-474 cells are also a highly invasive breast cancer cell line. More importantly, the BT-474 colonies are compact and can form multi-layered colonies on the MCF-10A feeder layer, unlike the MDA-MB-231 cells, which have a similar morphology as MCF-10A cells and never grow confluent (Figure 1).

The MCF-10A cells, for this purpose, were preincubated with pioglitazone and/or serum-rich growth media, at a specific concentration, for 1 week. The MCF-10A cells preincubated with pioglitazone and/or serum-rich growth media were then co-cultured adhesively and non-adhesively with BT-474 cells for another week. For the adhesive interaction, the BT-474 cells were grown on the feeder layer of MCF-10A cells that had been preincubated with pioglitazone and/or serum-rich growth media, whereas for the non-adhesive interaction, the BT-474 cells were incubated with the conditioned media of MCF-10A cells preincubated with pioglitazone and/or serum-rich growth media. The conditioned media were also used for ELISA immunoassays of soluble factors. Moreover, expression of soluble factors at the transcript level in the preincubated MCF-10A cells was determined using real-time PCR. The morphologies of MCF-10A cells and BT-474 cells before and after cell-cell interaction were examined using Oil-red O or Hemacolor Quick staining, and inverted microscopy. The study may provide useful information regarding the effects of pioglitazone and serum-rich growth medium on MCF-10A cells preincubation in which the preincubated MCF-10A cells are hypothesised to have growth inhibitory effects on breast cancer cells. The preincubated MCF-10A cells may have potential to be used as cell-mediated therapy for human breast cancers as well as for other malignancies.

## 2. Results and Discussion

### 2.1. The Viability of MCF-10A Cells Incubated with Pioglitazone-Containing Growth Medium

**Figure 2:**
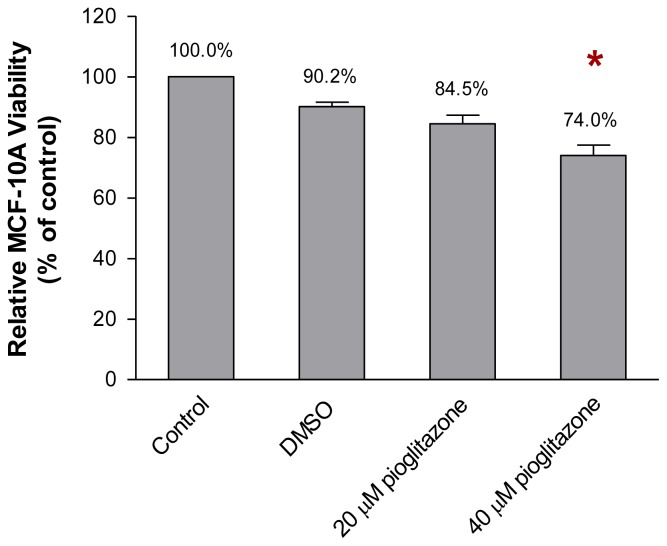
Viability of MCF-10A cells incubated with increasing concentrations of pioglitazone in the growth medium. One thousand cells were used as the input for incubation, and the incubation was carried out for 1 week. The values are expressed as mean ± SD from 3 replicates. * *p* < 0.05 was denoted as statistically significant.

Optimisation of the concentration of pioglitazone in the growth medium that modified the MCF-10A cells while allowing the MCF-10A cells to grow efficiently was performed, as described in Section 4.2. As shown in Figure 2, incubation of MCF-10A cells in the growth medium supplemented with 20 μM of pioglitazone for 1 week allowed 84.5% of the cells to grow, when compared to the cells which were incubated with growth medium only (control). However, increasing the concentration of pioglitazone in the growth medium to 40 μM significantly reduced the viability of the MCF-10A cells to 74.0% (*p* < 0.05), when compared to the control. This effect was only observed after the MCF-10A cells were incubated with the appropriate concentration of pioglitazone for 1 week. Thus, 20 μM, as determined in this preliminary study, was the optimum concentration of pioglitazone to be added to the growth medium to preincubate the MCF-10A cells for subsequent experiments.

### 2.2. The Growth of MCF-10A Cells Incubated with Serum-Rich Growth Medium

**Figure 3:**
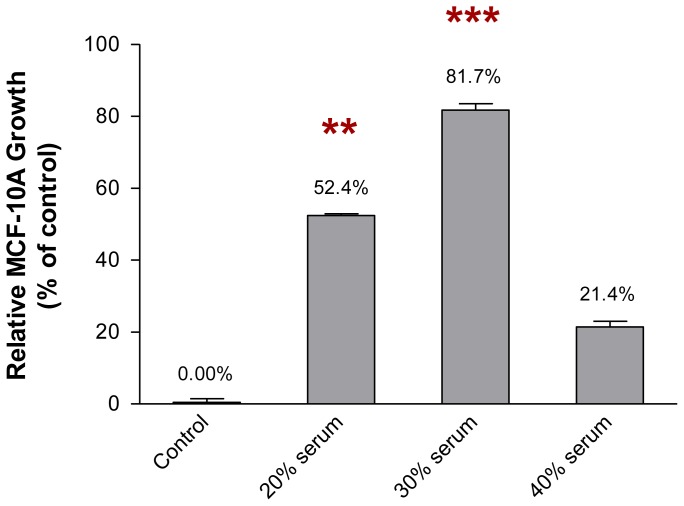
Growth of MCF-10A cells incubated with increasing concentrations of serum in the growth medium. One thousand cells were used as the input for incubation, and the incubation was carried out for 1 week. Each value was calculated as the % of incubated cells when compared to control cells. The values were expressed as mean ± SD from 3 replicates. ** *p* < 0.01 and *** *p* < 0.001 were denoted as statistically significant.

In contrast, Figure 3 shows the growth of MCF-10A cells in the growth medium supplemented with an increasing concentration of serum. Incubation of MCF-10A cells in the growth medium supplemented with different concentrations of serum for 1 week increased the growth of the cells. This finding was observed when the MCF-10A cells were incubated with the growth media supplemented with 20% and 30% of serum, where the growth of the cells was found to be 52.4% (*p* < 0.01) and 81.7% (*p* < 0.001), respectively, when compared to the cells incubated with growth medium only (control). However, the growth of the MCF-10A cells decreased to 21.4% when the cells were incubated with the growth medium supplemented with 40% of serum. Therefore, 30% of serum was the optimum concentration of serum to be added to the growth medium for the preincubation of MCF-10A cells. The growth media supplemented with 20 μM of pioglitazone and/or 30% of serum, which modified the MCF-10A cells while allowing the cells to grow efficiently, were used for the preincubation of MCF-10A cells. The growth media containing pioglitazone and/or serum, for the subsequent experiments, were then formulated as below: Culture Condition 1 as cells preincubated with growth medium only, Culture Condition 2 as cells preincubated with growth medium containing 30% of serum only, Culture Condition 3 as cells preincubated with growth medium containing 20 μM of pioglitazone only and Culture Condition 4 as cells preincubated with growth medium containing 30% of serum and 20 μM of pioglitazone. The MCF-10A cells were preincubated with respective culture condition for 1 week. The conditioned media of the preincubated MCF-10A cells was then collected and used for non-adhesive interaction study whereas the preincubated MCF-10A cells were harvested and used for soluble factor transcript expression analysis (Section 4.3).

### 2.3. The Morphology of MCF-10A Cells Preincubated with Pioglitazone and/or Serum-Rich Growth Media

**Figure 4:**
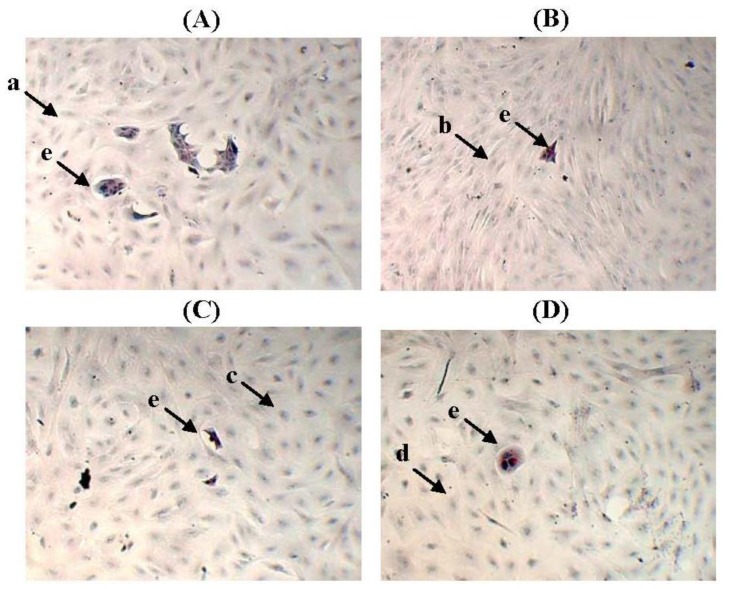
The morphologies of MCF-10A cells preincubated with (**A**) Culture Condition 1; (**B**) Culture Condition 2; (**C**) Culture Condition 3; and (**D**) Culture Condition 4. One hundred cells were used as the input for incubation, and the incubation was carried out for 1 week. The cells were stained with Oil Red O Staining. The pictures were taken with 200× magnification, where 0.01 mm was equal to 10 μm. Arrow **a**, **b**, **c** and **d** denote the MCF-10A cells preincubated with Culture Condition 1, 2, 3 and 4, respectively, whereas **e** denotes the BT-474 cells grown on the preincubated MCF-10A feeder layers.

Figure 4 shows the MCF-10A cells that were preincubated with pioglitazone and/or serum-rich growth media for 1 week. The preincubated MCF-10A cells showed little or no morphological changes, and no changes in aggregation status after the MCF-10A cells were preincubated with the different culture conditions, indicating that the preincubated MCF-10A cells may have undergone modification but were still able to grow efficiently in the cell culture conditions. This finding indicated that preincubating the MCF-10A cells with pioglitazone and/or serum-rich growth media did not harm the original structure of the cells. The MCF-10A cells looked more compact in Figure 4B were due to overlapping of the cells.

### 2.4. The Adhesive Interaction of BT-474 and MCF-10A Cells Preincubated with Pioglitazone and/or Serum-Rich Growth Media

**Figure 5:**
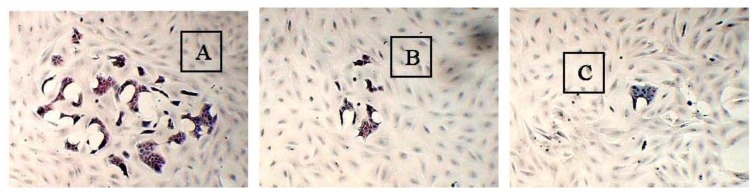
The BT-474 colonies on the feeder layer of MCF-10A cells preincubated with pioglitazone and/or serum-rich growth media. (**A**) Large BT-474 colonies in this study were denoted as a cell population of about 1–2 mm diameter on the preincubated MCF-10A feeder layer; (**B**) moderate BT-474 colonies were defined as a cell population of about 0.5 mm diameter on the preincubated MCF-10A feeder layer; and (**C**) small BT-474 colonies were defined as a cell population of less than 0.2 mm diameter on the preincubated MCF-10A feeder layer. The pictures were taken with 40× magnification, where 0.01 mm was equal to 10 μm.

The adhesive interaction study found that different size of BT-474 colonies formed on the feeder layer of MCF-10A cells that had been preincubated with pioglitazone and/or serum-rich growth media, after 2 weeks of incubation (Table 1). For this study, only large BT-474 colonies (100%) were grown on the feeder layer of MCF-10A cells that had been preincubated with Culture Condition 1 (Figure 5).

However, when the BT-474 cells were grown on the MCF-10A feeder layer that had been preincubated with Culture Conditions 2–4 for 2 weeks, the size of the BT-474 colonies (growth) was found to be smaller. The BT-474 cells that were grown on the feeder layer of MCF-10A cells preincubated with Culture Condition 2 for 2 weeks were observed to form about 30.0% large colonies, 20.0% moderate colonies and 50.0% small colonies on the preincubated MCF-10A feeder layer. Similarly, the BT-474 cells that were grown on the feeder layer of MCF-10A cells preincubated with Culture Condition 3 formed about 18.2% large cell colonies, 27.3% moderate cell colonies and 54.5% small cell colonies on the preincubated MCF-10A feeder layer. Surprisingly, a remarkable effect was found when the BT-474 cells were grown on the feeder layer of MCF-10A cells preincubated with Culture Condition 4, where only 20% moderate cell colonies and 80% small cell colonies on the preincubated MCF-10A feeder layer were observed. Few or no morphological changes, and no changes in aggregation status of the BT-474 cells were observed when the cells were grown on the feeder layer of MCF-10A cells preincubated with any of the tested culture conditions. The cancer cells were incubated for 2 weeks because the growth of the BT-474 cells on the preincubated MCF-10A feeder layer was only clearly and significantly observed after 2 weeks of incubation.

### 2.5. The Non-Adhesive Interaction of BT-474 Cells and MCF-10A Cells Preincubated with Pioglitazone and/or Serum-Rich Growth Media

**Figure 6:**
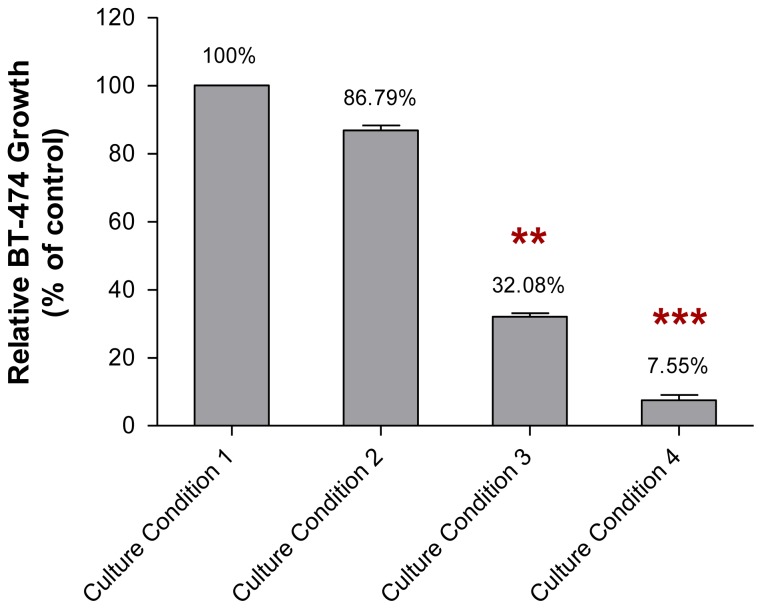
Growth of BT-474 cells incubated with the conditioned media of MCF-10A cells preincubated with Culture Condition 1, Culture Condition 2, Culture Condition 3 and Culture Condition 4. One hundred cells were used as the input for incubation, and the incubation was carried out for 2 weeks. Each value was calculated as the % of sample cells relative to the control cells. The values were expressed as mean ± SD from 3 replicates. ** *p* < 0.01 and *** *p* < 0.001 were denoted as statistically significant.

The non-adhesive interaction study found that the incubation of BT-474 cells with the conditioned media of MCF-10A cells preincubated with pioglitazone and/or serum-rich growth media for 2 weeks reduced the growth of the cancer cells (Figure 6). In this study, the growth of the BT-474 cells incubated with the conditioned medium of MCF-10A cells preincubated with Culture Condition 1 was denoted as 100%.

**Figure 7:**
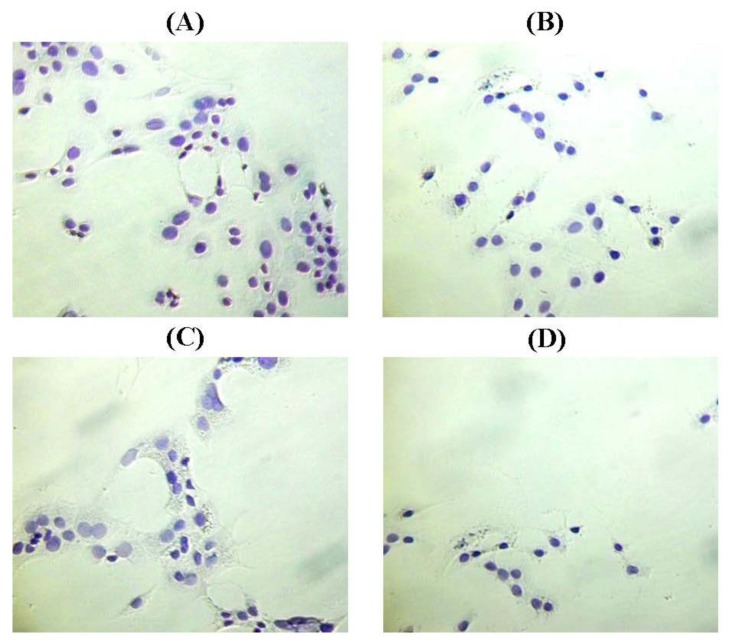
The morphologies of BT-474 cells preincubated with (**A**) Culture Condition 1; (**B**) Culture Condition 2; (**C**) Culture Condition 3; and (**D**) Culture Condition 4. One hundred cells were used as the input for incubation, and the incubation was carried out for 2 weeks. The cells were stained with Hemacolor Quick Staining. The pictures were taken with 200 × magnification, where 0.01 mm was equal to 10 μm.

The growth of BT-474 cells that were incubated with the conditioned media of MCF-10A cells preincubated with Culture Condition 2 and Culture Condition 3, for 2 weeks, was about 86.79% (*p* > 0.05) and 32.08% (*p* < 0.01), respectively, when compared to the growth of BT-474 cells incubated with the conditioned medium of MCF-10A cells preincubated with Culture Condition 1. In contrast, the growth of BT-474 cells incubated with the conditioned medium of MCF-10A cells preincubated with Culture Condition 4, for 2 weeks, was only about 7.55% (*p* < 0.001), when compared to the growth of BT-474 cells incubated with the conditioned medium of MCF-10A cells preincubated with Culture Condition 1. Moreover, similar to the adhesive interaction as mentioned above, despite the effect on the growth of the BT-474 cells, little or no morphological changes, and no changes in aggregation status were observed in the BT-474 cells when the cancer cells were incubated with the conditioned media of MCF-10A cells preincubated with any of the tested culture conditions (Figure 7). This finding indicated that the factors causing the BT-474 growth inhibition were likely presented in the medium and therefore could be detected in the conditioned media of MCF-10A cells preincubated with various culture conditions. These factors are likely affected only the growth of the BT-474 cells, but have little effect on the morphologies of both MCF-10A cells and BT-474 cells. Thus, the subsequent study was conducted to determine the levels of soluble factors existing in the conditioned media of preincubated MCF-10A cells using ELISA. These soluble factors are believed to have an impact on the cancer cell growth.

### 2.6. The Levels of Soluble Factors in the Conditioned Media of Preincubated MCF-10A Cells

As shown above, the non-adhesive interaction of BT-474 cells with MCF-10A cells that have been preincubated with pioglitazone and/or serum-rich growth media produced similar results as the studies of BT-474 cells interacting with the preincubated MCF-10A cells under adhesive conditions; both interactions reduced the growth of BT474 cells. This finding led us to hypothesise that the inhibition of BT-474 growth was likely due to the secretion of some soluble factors by the preincubated MCF-10A cells. These soluble factors that could be detected in the conditioned media of the preincubated MCF-10A cells were likely prohibited the BT-474 cells from growing. Therefore, it is important to determine the levels of the soluble factors (proteins) that are present in the conditioned media using ELISA. The soluble factors to be assayed in this study, soluble factors *a*–*d*, are reported to have proliferation-related effects on cancer cell growth, as reported in a few previous studies [6–13].

**Figure 8:**
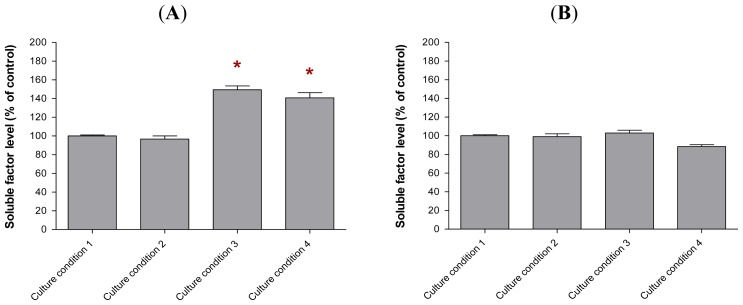

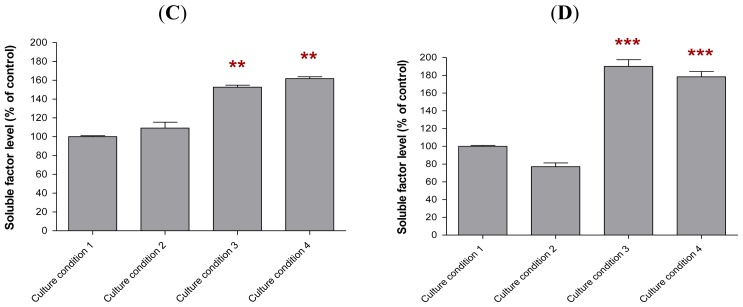
The expression levels of (**A**) soluble factor *a*, (**B**) soluble factor *b*, (**C**) soluble factor *c*, and (**D**) soluble factor *d* in the conditioned media of MCF-10A cells preincubated with pioglitazone and/or serum-rich growth media. The levels of the soluble factors were assayed by ELISA. The levels of soluble factors in the conditioned medium of MCF-10A cells preincubated with Culture Condition 1 was defined as 100%. The values were expressed as mean ± SD from 3 replicates. * *p* < 0.05, ** *p* < 0.01 and ** *p* < 0.001 were denoted as statistically significant.

Our study found that the soluble factors secreted by the preincubated MCF-10A cells in the conditioned media varied depending on the culture conditions used in this study (Figure 8). The same levels of soluble factors were detected in the conditioned media of MCF-10A cells preincubated with Culture Conditions 1 and 2, whereas elevated levels of soluble factor *a*, soluble factor *c* and soluble factor *d* were found in the conditioned media of MCF-10A cells preincubated with Culture Conditions 3 and 4. However, a slightly higher level of soluble factor *c* was only found in the conditioned media of MCF-10A cells preincubated with Culture Condition 4, thus displaying a difference from the Culture Condition 3. This elevated level of soluble factor *c* was likely resulted from the additional 30% of serum in the growth medium for the preincubation with Culture Condition 4 and caused better growth inhibitory effect on BT-474 rather than the use of pioglitazone alone. However, little or no change in the level of soluble factor *b* or CCL5 was found in the conditioned media of MCF-10A cells preincubated with all of the tested culture conditions, although the overexpression of CCL5 was reported to delay tumour growth as well as to increase tumour cell infiltration [14].

### 2.7. The Transcript Levels of Soluble Factors in the Preincubated MCF-10A Cells

**Figure 9:**
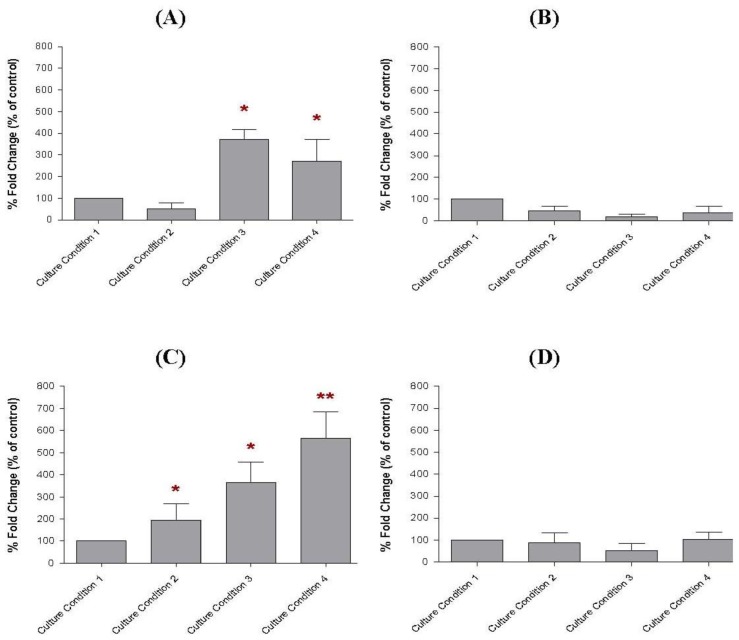
The transcript levels of (**A**) soluble factor *a*, (**B**) soluble factor *b*, (**C**) soluble factor *c* and (**D**) soluble factor *d* in MCF-10A cells preincubated with pioglitazone and/or serum-rich growth media. These genes’ transcripts were analysed using real-time PCR. Each value was calculated as a percentage of fold change in expression, in the preincubated MCF-10A cells. The gene transcript in the MCF-10A cells preincubated with Culture Condition 1 was defined as 100%. The values were expressed as mean ± SD from 3 replicates. * *p* < 0.05 and ** *p* < 0.01 were denoted as statistically significant.

Consistent with the ELISA results, similar levels of gene transcripts that correlated to those soluble factors in the conditioned media were also found in the MCF-10A cells preincubated with Culture Conditions 1–4, using real-time PCR (Figure 9). For this study, high expression level of soluble factor *a* transcript were found in the MCF-10A cells preincubated with Culture Conditions 3 and 4. These levels were 3.7-fold (*p* < 0.05) and 2.7-fold (*p* < 0.05) higher, respectively, when compared to the MCF-10A cells preincubated with Culture Condition 1. However, the soluble factor *a* transcript level in the MCF-10A cells preincubated with Culture Condition 2 was only 0.5-fold increased (*p* > 0.05), when the same comparison was made. The soluble factor *c* transcript was also found to be increased in the preincubated MCF-10A cells. The soluble factor *c* transcript levels in the MCF-10A cells preincubated with Culture Conditions 2, 3 and 4 were 2.0-fold (*p* > 0.05), 3.6-fold (*p* < 0.05) and 5.6-fold (*p* < 0.01) higher than the MCF-10A cells preincubated with Culture Condition 1, respectively. However, only the MCF-10A cells preincubated with Culture Condition 4 produced the most elevated soluble factor *c* transcript level, which differentiated the MCF-10A cells preincubated with this culture condition from the MCF-10A preincubated with Culture Condition 2 and Culture Condition 3. This phenomenon confirmed that the elevated level of soluble factor *c* was likely contributed by the additional 30% of serum in the growth medium for the preincubation and caused better growth inhibitory effect on BT-474 cells rather then the use of either serum-rich or pioglitazone alone in the growth medium. In contrast, while soluble factor *d* levels were found to be elevated in the conditioned media of preincubated MCF-10A cells, increased gene expression of soluble factor *d* was not detected in the preincubated MCF-10A cells using real-time PCR. The soluble factor *d*, in this study, is believed as VEGF. Consistently, little or no effect on the soluble factor *b* or CCL5 transcript was found in the preincubated MCF-10A cells in all culture conditions tested in this study.

## 3. Discussion

Our study demonstrated that (1) the adhesive interaction of BT-474 cells with the feeder layer of MCF-10A cells preincubated with pioglitazone and/or serum-rich media reduced the growth of the cancer cells, and (2) the non-adhesive interaction of BT-474 cells with the conditioned media of MCF-10A cells preincubated with pioglitazone and/or serum-rich media also reduced the growth of the cancer cells. The most promising inhibitory effect on BT-474 growth was observed when the cancer cells interacted adhesively and non-adhesively with the feeder layer or the conditioned medium of MCF-10A cells preincubated with serum-rich growth medium containing pioglitazone, which was followed by the feeder layer or the conditioned medium of MCF-10A cells that were incubated with pioglitazone-containing medium. As the adhesive and non-adhesive interaction studies of BT-474 cells with the preincubated MCF-10A cells produced a similar inhibitory effect on the cancer cells, this finding indicated that the inhibition of BT-474 growth caused by the preincubated MCF-10A cells can occur over some distance (medium) without the need to interact directly. Moreover, the inhibitory effect was likely caused by the secretion of certain soluble factors from the MCF-10A cells into the conditioned medium after the preincubation, where the soluble factors were believed to prohibit the BT-474 cells from growing. These soluble factors could be detected by ELISA in the conditioned media of the preincubated MCF-10A cells.

The ELISA immunoassays showed that the conditioned media of MCF-10A cells that had been preincubated with growth medium containing pioglitazone alone or with a combination of pioglitazone and serum-rich growth medium secreted higher levels of soluble factor *a*, soluble factor *c* and soluble factor *d* into the conditioned media. These soluble factors in the conditioned media were likely inhibited the growth of BT-474 cells when the cancer cells interacted adhesively or non-adhesively with the preincubated MCF-10A cells. The preincubated MCF-10A cells secreted soluble factor *a*, soluble factor *c* and soluble factor *d* into the conditioned media were likely increased survival of the MCF-10A cells against the growth of the cancer cells. Besides, high levels of soluble factor *a* and soluble factor *c* transcripts were also detected in the MCF-10A cells preincubated with pioglitazone and/or serum-rich growth media using real-time PCR. Although the soluble factor *a* and soluble factor *c* were over-expressed in the preincubated MCF-10A cells, only a higher level of soluble factor *c* was found in the MCF-10A cells preincubated with serum-rich growth medium containing pioglitazone. This phenomenon implicated the secretion of high level of soluble factor *c* as the factor causing the most promising inhibitory effect on BT-474 growth when the cancer cells interacted with the feeder layer or the conditioned medium of the MCF-10A cells that were incubated with serum-rich growth medium containing pioglitazone than that with the feeder layer or the conditioned medium of MCF-10A cells that were incubated with pioglitazone-containing growth medium only. The soluble factor *a* and soluble factor *c* that were overexpressed in the conditioned media of the preincubated MCF-10A are believed as CCL2 and IL-6, respectively.

CCL2, also known as MCP-1, is a CC-chemokine that is chemotactic for monocytes, memory T-cells and NK cells [15,16]. It is expressed by a wide variety of cancer types. A large number of studies have demonstrated that CCL2 generally facilitates tumour progression where this phenomenon can be observed in pancreatic [17], breast [18,19] and gastric cancers [20]. However, our present study demonstrated that an increased level of CCL2 inhibited the growth of BT-474 cells, paving a new discussion on this topic. In addition, IL-6 is an interleukin that plays a key role in the pathophysiology of several cancers and various inflammatory disorders of the immune system [21]. Its over-expression has been implicated in the tumourigenesis of multiple myeloma, ovarian, renal cell, prostate, cervical and breast carcinomas [22–25]. In a previous study, CCL2 was hypothesised to contribute to the development of lung fibrosis by reducing IL-6 level [26]. However, the precise mechanisms of action of both CCL2 and IL-6 in the preincubated MCF-10A cells are not yet elucidated. As the preincubated MCF-10A cells may have potential to be used for cell-mediated therapy, both CCL2 and IL-6 secreted by the preincubated MCF-10A cells should carefully be analysed in our future research. Perhaps, novel finding, such as new CCL2 and IL-6 isoforms, may be identified in this part of the study.

For the first time, this study found that the over-expression of CCL2 and IL-6 in the conditioned medium of the preincubated MCF-10A cells can reduce the growth of BT-474 cells. However, the precise anti-tumoural effect of both CCL2 and IL-6 on BT-474 growth remains unclear. Perhaps, there are some additional unknown factors in the conditioned media that inhibit the growth of BT-474 cells. CCL2, which stimulates IL-6 production, may contribute to the development of fibrosis in the preincubated MCF-10A cells and should also be taken into account [26]. Taken together, the interaction of the preincubated MCF-10A cells with BT-474 cells only reduced the growth of the cancer cells. Little or no morphological changes and no changes in the aggregation status of both the MCF-10A and BT-474 cells were observed during the interaction. This work may provide useful information about the factors that may prevent the tumorigenic cells from growing on the non-tumourigenic feeder layer or that may prevent the metastatic cancer cells from migrating to the non-tumourigenic tissue or organ. The MCF-10A cells that have been preincubated with pioglitazone and/or serum-rich growth media may have potential to be used as a component for cell-mediated therapy for human breast cancers as well as for other malignancies. For example, the preincubated MCF-10A cells, that exhibited anti-cancer growth properties, may be injected in the mammary fat pad in order to treat the cancer patients or to be used for others cell-mediated therapy. Although the challenges facing how the modified MCF-10A cells can be injected in the human body are formidable, it is likely possible. We are aware also of the concern that the preincubated cells and the derived tissue would be rejected by the patient's immune system. However, based on current proposed solutions to this incompatibility problem worldwide, such as generating large banks of stem cell lines that perfectly match the patient or genetically engineering stem cells to reduce immune rejection, it may provide essentially convincing evidence and prove the complex scientific endeavor. A similar approach has been utilized by AdiStem Ltd., a biotechnology company, to repair tissues in the human body. For this, adipose tissue-derived stem cells from a patient are treated and activated with AdiStem solution (activator). The activated stem cells are then returned to the patients *via* a standard IV drip.

## 4. Experimental Section

### 4.1. Culturing of MCF-10A and BT-474 Cell Lines

The MCF-10A cell line was routinely cultured with growth medium for non-tumourigenic human breast cells (low glucose Dulbecco’s Modified Eagle’s Medium (DMEM) supplemented with 10% fetal bovine serum (FBS), 100 U/mL penicillin and 100 mg/mL streptomycin with stable glutamine and Na-pyruvate), whereas the BT-474 cell line was cultured using the growth medium for tumourigenic human breast cells (high glucose DMEM supplemented with 10% FBS, 100 U/mL penicillin and 100 mg/mL streptomycin). An optional supplement of 1× Mycokill was added to both growth media to prevent mycoplasma contamination. The cell lines were maintained at 37 °C in a humidified atmosphere of 5% (*v*/*v*) CO_2_. The growth media for MCF-10A cells and BT-474 cells were changed every 2–3 days. The cell lines were sub-cultured and maintained for subsequent experiments.

### 4.2. Viability of MCF-10A Cells Incubated with Pioglitazone-Containing or Serum-Rich Growth Medium

The viability of MCF-10A cells incubated with either pioglitazone-containing or serum (FBS)-rich growth medium was determined using trypan blue assay. The viability of the cells was determined in order to select the appropriate concentrations of pioglitazone and serum with which to preincubate the MCF-10A cells. For this assay, the MCF-10A cells were seeded at a density of 1.0 × 10^3^ cells/mL in a 24-well culture plate. When the cells in each well reached approximately 70% confluency, increasing concentrations of pioglitazone (0–40 μM) or serum (10–40%) were added to the cells. The cells were then incubated in the culture conditions, as described above, for 1 week. DMSO (0.1%) was used as the diluent control for pioglitazone in this study. All control and experimental samples were cultured in triplicate. After 1 week, the cells were harvested using trypsin-EDTA solution. The harvested cell suspension (0.9 mL) from each well was then combined with 0.1 mL of Trypan Blue Solution, and the mixture was incubated at room temperature for 5 min. Living and dead cells were then counted using a haemocytometer and the viability (%) of the MCF-10A cells was calculated as below:

(i) Viability (%)=(number of unstained cells per quadrant/total cells per quadrant)×100

Living cells were observed to exclude the dye (unstained cells), whereas dead cells took up the blue dye. The same method was used to calculate the viability of MCF-10A cells preincubated with growth media supplemented with 10–40% serum. The appropriate concentrations of pioglitazone and serum were selected so that their presence in the media modified the MCF-10A cells, but did not significantly decrease the viability of the MCF-10A cells. The concentrations of pioglitazone and serum used in this study were optimised and selected as described in Section 2.1 and 2.2. After that, the pioglitazone-containing and serum-rich growth media used to preincubate the MCF-10A cells was prepared, as described in Table 2.

### 4.3. Preparation of the Conditioned Media and MCF-10A Cells Preincubated with Pioglitazone and/or Serum-Rich Growth Media

**Figure 10:**
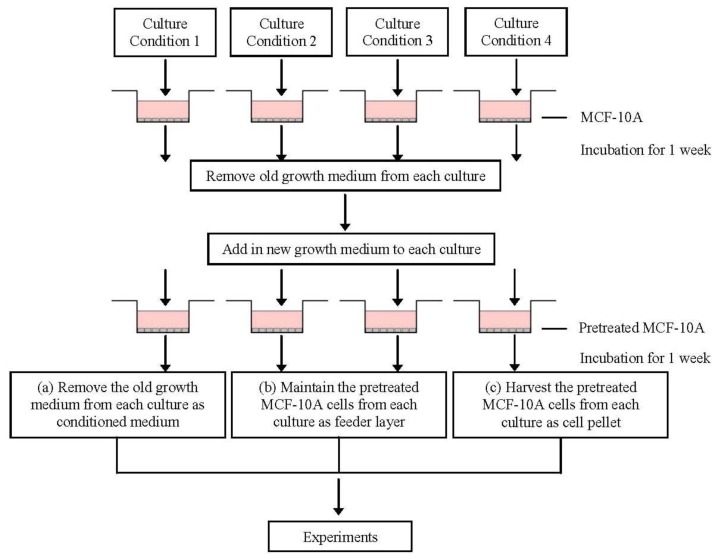
Schematic of the conditioned media and MCF-10A cells preincubated with pioglitazone and/or serum-rich growth media preparations.

The conditioned media and MCF-10A cells preincubated with pioglitazone and/or serum-rich growth media were prepared by seeding 1.0 × 10^4^ cells/mL of MCF-10A cells in T-75-cm^2^ cell culture flasks and the cells were maintained with low glucose DMEM growth medium, as described above, until the cells were 70–80% confluent. Next, the old growth medium was removed and the adherent MCF-10A feeder layer was carefully washed several times with prewarmed PBS before adding the appropriate concentration of pioglitazone and/or serum-rich supplemented growth media, as described in Table 2. The MCF-10A cultures were then incubated under the appropriate culture conditions for 1 week. After 1 week of incubation, the pioglitazone and/or serum-rich supplemented growth media were removed, and the preincubated MCF-10A feeder layer, for all culture conditions, was carefully washed again several times with prewarmed PBS before fresh growth media were added to the MCF-10A feeder layer. The preincubated MCF-10A feeder layer was then incubated with the fresh growth medium again for 1 week. Following a week of incubation, the growth media, which is now described as conditioned media, for all culture conditions was collected in 15 mL falcon tubes. The conditioned media was centrifuged at maximum speed for 20 min to concentrate any particles or cell debris, and the supernatants were collected and used for non-adhesive interaction study with BT-474 cells, as described in Section 4.5, or were used for immunoassay, as described in Section 4.6. The preincubated MCF-10A cells were also monitored using Hemacolor Quik staining and inverted microscopy, or harvested for soluble factor transcript expression study, as described in Section 4.7. In brief, the preparations of the conditioned media and preincubated MCF-10A cells are outlined in Figure 10.

### 4.4. Adhesive Interaction of BT-474 Cells and MCF-10A Cells Preincubated with Pioglitazone and/or Serum-Rich Growth Media

The adhesive interaction or direct co-culture of BT-474 cells with MCF-10A cells preincubated with pioglitazone and/or serum-rich growth media was carried out by seeding 1.0 × 10^3^ MCF-10A cells/mL in a 4-well chamber slide. The cells were allowed to adhere overnight. The MCF-10A cells were then preincubated with pioglitazone and/or serum-rich growth media as described in Section 4.3. After 1 week, the growth media supplemented with pioglitazone and/or serum was removed, the preincubated MCF-10A feeder layer was carefully washed and a suspension of BT-474 cells (10 cells/well) was added to the preincubated MCF-10A feeder layer. The BT-474 cells were also cultured alone as control. After 2 weeks of incubation, the morphology and the number of BT-474 colonies that grew on the preincubated MCF-10A cells were monitored and quantified using Oil Red O staining and inverted microscopy. The co-culture was repeated at least twice.

### 4.5. Non-Adhesive Interaction of BT-474 Cells with MCF-10A Cells Preincubated with Pioglitazone and/or Serum-Rich Growth Media

The non-adhesive interaction or indirect co-culture of BT-474 cells with MCF-10A cells preincubated with pioglitazone and/or serum-rich growth media was performed by incubating the BT-474 cells with the conditioned media of preincubated MCF-10A cells collected from Culture Conditions 1–4, as described in Section 4.3. The BT-474 cells, for this experiment, were seeded (1.0 × 10^2^ cell/mL) and maintained with low glucose DMEM growth medium in a 12-well plate. After 24 h of incubation, the old growth medium was removed and the conditioned media from Culture Conditions 1–4, was collected as described in Section 4.3, and added to the BT-474 cells. The BT-474 cells were incubated in conditioned media for 2 weeks. Incubation of the BT-474 cells with each conditioned medium was performed in triplicate. After 2 weeks incubation, the BT-474 cells were trypsinised, and the viability of the cells was quantified using the trypan blue exclusion assay. The co-culture was repeated at least twice.

### 4.6. Immunoassay of Soluble Factors in the Conditioned Media

The levels of soluble factors *a*–*d* present in the conditioned media of preincubated MCF-10A cells were determined using commercially available ELISA kits. In these kits, antibodies specific for the proteins were precoated onto microtitre plates. The samples (conditioned media) were then added to the wells and allowed to react with the bound antibody for 2.5 h at room temperature. The unbound substances were washed away with a Wash Solution, according to the manufacturer’s instruction. Subsequently, an enzyme-linked antibody specific to the proteins was added to the wells. The antibody was incubated with the target for 1 h. Following another washing step, a substrate solution was added to the wells for colour development. The colour developed was proportional to the amount of protein present in the samples. The colour intensity was then measured using an ELISA reader at a wavelength of 450 nm, and the level of each specific protein in the conditioned media was calculated. The ELISA were performed in triplicate and repeated in at least 2 independent experiments.

### 4.7. Analysis of Soluble Factor Transcript Expressions in the Preincubated MCF-10A Cells

The total RNA was extracted from the preincubated MCF-10A cells using a commercially available total RNA extraction kit. The integrity of the total RNA extracted was confirmed by agarose gel electrophoresis, and the purity and concentration of the total RNA extracted was measured by spectrophotometry. Subsequently, 1.0 μg of RNA was reverse-transcribed into cDNA using a commercially available First Strand cDNA Synthesis kit, and this cDNA was used to analyse the expressions of soluble factor transcripts in the preincubated MCF-10A cells by real-time PCR. Primers specific to these genes were designed using Primer Express 2.0 (Table 3), and the real-time PCR was performed using a Rotor Gene 600 PCR system, according to the manufacturer’s instruction. To do this, the reactions were carried out in a total volume of 25 μL, in optical reaction tubes that included SYBR Green Master Mix, 900 nM of each primer and cDNA as prepared above. The reaction program was then initiated at 95 °C for 10 min to activate the enzyme, follow by 40 cycles of denaturation at 95 °C for 15 s, and primer annealing combined with extension stage was performed at 60 °C for 1 min. The CT value of each gene in each unknown sample was normalised to β-actin, and the relative expression level of each gene, as well as the fold changes in gene expression, were calculated, according to the manufacturer’s instruction. The PCR was performed in triplicate and repeated in at least 2 independent experiments.

### 4.8. Statistical Analysis

All graphs and all statistical calculations were generated and performed using *GraphPad* 4.01 software. The experiments were repeated several times to confirm the reproducibility of the results. All values are expressed as the mean ± SD. A *p* value less than 0.05 was regarded as statistically significant.

## 5. Conclusions

Our study has demonstrated that the MCF-10A cells that have been preincubated with pioglitazone and/or serum-rich growth media for 1 week were able to reduce the growth of BT-474 cells when the preincubated MCF-10A cells and BT-474 cells interacted with one another adhesively or non-adhesively. This reduction was likely due to the overexpression of some soluble factors in the preincubated MCF-10A cells. However, future studies are needed to understand the precise mechanisms of action for the soluble factors in the preincubated MCF-10A cells as well as the anti-tumoural effects of the preincubated MCF-10A cells on the growth of BT-474 cells.

## Figures and Tables

**Table 1:** Percentage of BT-474 colonies grown on the feeder layer of MCF-10A cells preincubated with pioglitazone and/or serum-rich growth media for 2 weeks. A total of 20 cells/mL of BT-474 was used as the input for all culture conditions. Data were calculated as the % of colony number, according to the size, per total number of colonies formed on the preincubated MCF-10A feeder layer. The experiments were repeated 3 times to confirm reproducibility of the results, and a representative result is shown below.

Culture Condition	Large cell colony	Moderate cell colony	Small cell colony
**1 (control)**	100.0%	-	-
**2**	30.0%	20.0%	50.0%
**3**	18.2%	27.3%	54.5%
**4**	-	20.0%	80.0%

**Table 2:** Incubation of MCF-10A cells with low glucose DMEM growth media supplemented with pioglitazone and/or serum at the appropriate concentrations, as described in the Table, for 1 week. The concentrations of pioglitazone and serum used in this study were optimised as described in the results section.

Culture Condition	Serum Concentration	Pioglitazone Concentration
1 (control)	10% *	-
2	30%	-
3	10% *	20 μM
4	30%	20 μM

* The FBS from growth medium only.

**Table 3:** Primer design for real-time PCR.

Gene(s)	Sequence(s)	Amplicon (bp)	*T*_m_ (°C)
Soluble factor *a*	Forward: 5′-GCTGTGATCTTCAAGACCATTGTG-3′	123	80
	Reverse: 5′-AGTGAGTGTTCAAGTCTTCGGAGTT-3′		
Soluble factor *b*	Forward: 5′-AGCCTCTCCCACAGGTACCAT-3′	67	85
	Reverse: 5′-GGCAGTAGCAATGAGGATGACA-3′		
Soluble factor *c*	Forward: CCAGTACCCCCAGGAGAAGATT	104	81
	Reverse: CCGTCGAGGATGTACCGAAT		
Soluble factor *d*	Forward: 5′-CGCAGCTACTGCCATCCAAT-3′	81	81
	Reverse: 5′-TGGCTTGAAGATGTACTCGATCTC-3′		
β-actin	Forward: 5′-CATTGCCGACAGGATGCA-3′	102	82
	Reverse: 5′-CCGATCCACACGGAGTACTTG-3′		
